# Awareness of Foodborne Pathogens among Students: A Cross-Sectional Study in the Kingdom of Saudi Arabia

**DOI:** 10.1155/2021/9971748

**Published:** 2021-05-22

**Authors:** Mohammed AL-Mohaithef

**Affiliations:** Department of Public Health, College of Health Sciences, Saudi Electronic University, Riyadh, Saudi Arabia

## Abstract

Food poisoning associated with microbial pathogens causes millions of foodborne disease cases in the Kingdom of Saudi Arabia. Awareness about foodborne pathogens may play a positive role in the reduction of foodborne illnesses. The present cross-sectional study investigates awareness about five major microbial pathogens (*Salmonella*, *Campylobacter*, *Staphylococcus*, *Listeria*, and *Escherichia coli*) among university students (*n* = 399) from four major cities in the Kingdom of Saudi Arabia. Multiple logistic regression models were used to predict the determinants of inadequate knowledge. Of the 399 study participants, only 34.5% of students knew the above-mentioned foodborne pathogens. Awareness varied by pathogen, and the variations appeared to be related to age, sex, education, and field of study. In comparison to students in health sciences, students in computer sciences were found to be less knowledgeable about foodborne pathogens (OR: 2.85; 95% CI: 1.36-5.99). Our findings suggest that awareness about microbial pathogens is low among students and is associated with their field of study. Effective education programs about foodborne hazards could help improve students' awareness of microbial pathogens.

## 1. Introduction

Microbial contamination of food is a major public health concern due to the emergence of foodborne pathogens [1, 2]. More than 250 sources of foodborne diseases have been identified globally [3]. Numerous studies indicate that foodborne outbreaks are becoming more frequent globally and have urged public health interventions [4–7]. Each year, microbial pathogens cause millions of foodborne disease cases, resulting in many hospitalizations and deaths in Saudi Arabia [8]. The highest proportion of outbreaks was in adults (68.1%), and *Salmonella* species (81%) was the most common causative agent, followed by *Staphylococcus aureus* (19%) [9]. In the United States, 31 major pathogens caused 9.4 million foodborne illnesses [10]. Most (58%) illnesses were caused by norovirus, followed by nontyphoidal *Salmonella* spp. (11%), *Clostridium perfringens* (10%), and *Campylobacter* spp. (9%). Between 1964 and 2010, listeriosis cases occurred in 28 (90%) provinces in China, and the overall case-fatality rate was 26% [11]. Due to the increase in foodborne infectious diseases, several food quality regulations have been implemented in various countries [11–16]. Foodborne pathogens reduce human health productivity and pose a substantial economic cost to the health system [17, 18]. However, there is a knowledge gap on awareness of microbial pathogens in food, as reported by a recent systematic review and meta-analysis [19]. Food safety efforts generally focus on laboratory examination and physical investigation of the end product, while interventions to improve knowledge on the causes of foodborne illness have received limited attention.

A study in the United States reported low awareness of four major microbial pathogens (*Salmonella*, *Campylobacter*, *Listeria*, and *E. coli*) among consumers and reported associations with demographics and food safety behaviors [20]. In Jordan, only 65.1% of the study participants knew about foodborne disease. Only 23.1% knew that the presence of *Shigella* in food can cause illness, and 50.1% have heard of salmonellosis [21]. In the Kingdom of Saudi Arabia, only 49% of consumers have information about *Salmonella* as a foodborne pathogen. In comparison, only 16% mentioned *Campylobacter* as a foodborne pathogen [22]. Foodborne illnesses are prevalent in Saudi Arabia, but the number of infection and associated deaths has not been accurately reported. Previous studies have suggested that awareness of foodborne pathogens is related to a better knowledge of safe food handling and preparation principles [23]. A recent study highlighted that consumers with more awareness of foodborne pathogens were more likely to adopt food safety practices [24]. Therefore, evaluating foodborne pathogen knowledge among young individuals is important, as interventions can be implemented to help them develop proper attitudes and skills to understand contemporary food issues to improve public health. The study was aimed at assessing students' knowledge about five major foodborne pathogens and to predict their knowledge with their sociodemographic profile in Saudi Arabia.

## 2. Materials and Methods

### 2.1. Study Design and Participants

A cross-sectional study was designed among registered students at Saudi Electronic University in four significant cities (Abha, Dammam, Jeddah, and Riyadh) in the Kingdom of Saudi Arabia. Five hundred random students were contacted to participate in the study; however, only 399 students participated in the survey (79.8% response rate).

### 2.2. Data Collection

After obtaining informed consent, students were asked to complete the survey. The questionnaire of each respondent was coded to ensure anonymity. Data were collected through interviews by trained enumerators using a validated questionnaire. The questionnaires were completed directly by the respondents. Each questionnaire took approximately 10 to 20 minutes to complete.

### 2.3. Data Analysis

Statistical analysis of the data was performed using STATA software (version 13.0). Descriptive statistics were used to generate summary statistics. Pearson's chi-squared test was used to investigate the association between sociodemographic differences in foodborne pathogen knowledge among students. Logistic regression models were constructed to predict the association between students' knowledge and their sociodemographic profile. Statistical significance was identified at the 95% confidence level (*p* < 0.05).

### 2.4. Ethical Considerations

The study protocol was reviewed and approved by the Institutional Review Board of Saudi Electronic University. Study participants were asked to participate voluntarily and allowed to withdraw from the study if they so desired. Survey results were anonymized and analyzed for interpretation.

## 3. Results

The sociodemographic characteristics of the study participants are presented in Table 1. Of the 399 study participants, approximately 29.5% resided in the city of Abha, followed by Riyadh (29.0%), Dammam (24.3%), and Jeddah (17.0%). More than half (55.14%) of the respondents were female. Approximately 25.0% of the students were younger than 20 years, with the majority falling in the 20–30-year range. A majority of the respondents (78.70%) studied at the bachelor's level of a university program, and more than half (58.7%) of the respondents were enrolled in the health sciences program.

Table 2 represents the association between students' knowledge of foodborne pathogens and their sociodemographic characteristics. The overall knowledge of foodborne pathogens among students was found to be poor. Only 34.5% of students recognized all five major foodborne pathogens listed in this study. Of the 399 students, only 88 (22%), 43 (10.7%), and 60 (15%) were aware of *E. coli*, *Campylobacter*, *Staphylococcus*, and *Listeria*, respectively. Approximately 60% of students were aware of *Salmonella* as a foodborne pathogen. Knowledge of *Salmonella* was found to be associated with sex (*p* = 0.020), education (*p* ≤ 0.001), field of study (*p* ≤ 0.001), and place of residency (*p* = 0.002).

The multiple logistic regression model results suggest that age, sex, and field of study are significant determinants in explaining the pathogen knowledge variables in the study (Table 3). Female students were found to be more knowledgeable (OR: 0.46; 95% CI: 0.21-0.99). In comparison to students in the health sciences, students in the fields of computer sciences (OR: 2.85; 95% CI: 1.36-5.99), administration and business (OR: 1.98; 95% CI: 0.93-4.24), and languages and theoretical studies (OR: 3.62; 95% CI: 1.68 -7.78) were found to be less knowledgeable about foodborne pathogens.

## 4. Discussion

This study revealed that the majority (65%) of student respondents were not aware of foodborne pathogens. Only 34.5% of students recognized all the major foodborne pathogens listed in this study: only 88 (22%), 43 (10.7%), 60 (15%), and 39 (9.8%) were aware of *E. coli*, *Campylobacter*, *Staphylococcus*, and *Listeria,* respectively. *Salmonella* was a well-known pathogen among our study participants, and similar findings were reported in other studies [25]. Our study shows that there is a need for health education programs for students, especially those emphasizing foodborne pathogens. A similar study conducted in Taif, Kingdom of Saudi Arabia, revealed the lack of knowledge among people on foodborne pathogens. In that study, only half of the participants knew about salmonellosis, the most commonly known cause of food poisoning, 27% had information about hepatitis, and 22% had knowledge of *E. coli* [22]. Similarly, a study from Jordan showed that 76.9% (three-fourths) of the respondents had not heard of Shigellosis and did not know that it could be transmitted to humans through food [21]. A review study from Saudi Arabia highlights the magnitude and determinants of food poisoning internationally and in the Kingdom [8]. Another review pointed out that older adults' awareness of *Listeria* varies between 33 and 58%, with a median understanding of only 40% [19].

Compared to previous studies, our study findings indicate the following important points about pathogen awareness among students in the Kingdom of Saudi Arabia. First, more students said they had heard of *Salmonella* (60.15%) than the other foodborne pathogens. Second, students' with a health science background were significantly more informed about *Salmonella* (70%) than students with other backgrounds. Third, male students (40.42%) were less informed about foodborne pathogens than female students. Our study findings suggest that awareness of different pathogens (*Salmonella*, *Campylobacter*, *Staphylococcus*, *Listeria*, and *Escherichia coli*) among students is related to their field of study. As reported, health science students are more aware than their peers in other programs of study. This may also suggest that health science students have better access to microbial food safety information. Female students are more knowledgeable about pathogens than their male counterparts. This may be because of their food handling behavior and access to food safety information.

Furthermore, students are a known high-risk population for foodborne illness due to their high-risk eating behavior [21, 26–28]; imparting to them the knowledge of pathogen-specific unsafe food sources and prevention practices may help in changing their behaviors. As suggested by numerous studies, our findings also highlight that promoting understanding of microbial pathogens in food to students is necessary. It could be an essential intervention in reducing the burden of foodborne disease in Saudi Arabia.

## 5. Limitations

Our study cannot rule out other confounding variables in predicting students' awareness levels. One of the study's limitations is that it measured self-reported knowledge on foodborne pathogens, and respondents could over/underreport their understanding of pathogens. Moreover, the study is focused on students in university settings and cannot be generalized.

## 6. Conclusions

Considering the results, it is concluded that the study participants in Saudi Arabia were poorly informed about major foodborne pathogens (*Salmonella*, *Campylobacter*, *Staphylococcus*, *Listeria*, and *Escherichia coli*). Among the five pathogens, students were more informed about *Salmonella* and showed a lower level of awareness of other pathogens in food. There is a need to conduct food safety education programs for university students to improve their level of awareness about foodborne pathogens.

## Figures and Tables

**Table 1:** Demographic characteristics of study participants (*N* = 399).

Variables	*n*	%
*Age*		
Less than 20	106	26.57
20-25	95	23.81
26-30	104	26.07
Above 30	94	23.56

*Sex*		
Male	179	44.86
Female	220	55.14

*Educational level*		
Preparatory year	72	18.05
Bachelor	314	78.70
Master	13	3.26

*Field of study (specialization)*		
Health sciences	252	58.7
Computer sciences	44	10.3
Languages and theoretical studies	52	12.1
Administration and business	56	13.1
Others	25	5.8

*Place of residence (city)*		
Abha	118	29.57
Dammam	97	24.31
Jeddah	68	17.04
Riyadh	116	29.07

**Table 2:** Association of students' knowledge of foodborne pathogens with their sociodemographic characteristics (*N* = 399).

Factors	Knowledge of foodborne pathogens^+^*n* = 138 (34.58)	Students recognized at least one of the following foodborne pathogens				
		*E. coli* *n* = 88 (22.05)	*Campylobacter* *n* = 43 (10.77)	*Staphylococcus* *n* = 60 (15.03)	*Listeria* *n* = 39 (09.77)	*Salmonella* *n* = 240 (60.15)
Age						
Above 30	27 (19.57)	20 (22.73)	12 (27.91)	11 (18.33)	05 (12.82)	63 (26.25)
30-26	33 (23.91)	32 (36.36)	13 (30.23)	23 (38.33)	10 (25.64)	64 (26.27)
25-20	36 (26.09)	15 (17.05)	05 (11.63)	10 (16.67)	10 (25.64)	57 (23.75)
Less than 20	42 (30.43)	21 (23.86)	13 (30.23)	16 (26.67)	14 (35.90)	56 (23.33)
*Pearson chi (pvalue)*	*0.327*	*0.069*	*0.266*	*0.093*	*0.308*	*0.230*

Sex						
Male	70 (50.72)	44 (50.0)	19 (44.19)	34 (56.27)	15 (38.46)	97 (40.42)
Female	68 (49.28)	44 (50.0)	24 (55.81)	26 (43.33)	24 (61.54)	143 (59.58)
*Pearson chi (pvalue)*	*0.087*	*0.272*	*0.925*	*0.046*∗	*0.397*	*0.020*∗

Educational level						
Master	03 (2.17)	07 (8.51)	03 (06.98)	03 (05.00)	03 (07.69)	09 (03.75)
Bachelor	91 (65.94)	73 (8.51)	32 (74.42)	54 (90.00)	28 (71.79)	209 (87.08)
Preparatory year	44 (31.88)	08 (8.51)	08 (18.60)	03 (05.00)	08 (20.51)	22 (09.17)
*Pearson chi (pvalue)*	*≤0.001*∗	*0.002*∗	*0.339*	*0.015*∗	*0.221*	*≤0.001*∗

Field of study						
Health sciences	52 (37.68)	69 (8.51)	30 (69.77)	51 (85.00)	27 (69.23)	168 (70.00)
Computer sciences	17 (12.32)	04 (8.51)	00 (00.00)	04 (06.67)	03 (07.69)	22 (9.17)
Administration and business	24 (17.39)	09 (8.51)	07 (16.28)	05 (8.33)	05 (12.82)	24 (10.10)
Languages and theoretical studies	28 (20.29)	03 (8.51)	04 (09.20)	00 (0.00)	04 (10.26)	22 (9.17)
Others	17 (12.32)	03 (8.51)	02 (04.65)	00 (0.00)	00 (0.00)	04 (1.67)
*Pearson chi (pvalue)*	*≤0.001*∗	*≤0.001*∗	*0.146*	*≤0.001*∗	*0.439*	*≤0.001*∗

Place of residence (city)						
Riyadh	41 (29.71)	21 (8.51)	10 (23.26)	09 (15.00)	09 (23.08)	69 (28.75)
Abha	52 (37.68)	21 (8.51)	11 (25.58)	21 (35.00)	07 (17.95)	58 (24.17)
Dammam	21 (15.22)	33 (8.51)	17 (39.53)	21 (35.00)	16 (41.03)	73 (30.42)
Jeddah	24 (17.39)	13 (8.51)	05 (11.63)	09 (15.00)	07 (17.95)	40 (16.67)
*Pearson chi (pvalue)*	*0.008*∗	*0.013*∗	*0.100*	*0.029*∗	*0.058*	*0.002*∗

^+^Students able to recognize all major foodborne pathogens listed in this study. ^∗^Statistically significant (*p* < 0.05).

**Table 3:** Results of logistic regression for unadjusted and adjusted odds ratios for association between student's poor^+^ knowledge of foodborne pathogens and their sociodemographic characteristics (*N* = 399).

Factors	Unadjusted OR [95% CI]	Adjusted OR [95% CI]	*p* value
*Age*			
Above 30	1.00	1.00	
30-26	1.15 [0.62-2.11]	2.23 [1.13-4.41]	0.020^∗^
25-20	1.51 [0.82-2.78]	1.18 [0.60-2.31]	0.629
Less than 20	1.62 [0.90-2.94]	3.04 [1.47-6.30]	0.003^∗^

*Sex*			
Male	1.00	1.00	
Female	0.69 [0.46-1.05]	0.46 [0.21-0.99]	0.048^∗^

*Educational level*			
Master	1.00	1.00	
Bachelor	1.36 [0.36-5.05]	1.96 [0.41-9.31]	0.394
Preparatory year	5.23 [1.32-20.70]	1.17 [0.27-5.06]	0.831

*Field of study*			
Health sciences	1.00	1.00	
Computer sciences	2.70 [1.33-5.46]	2.85 [1.36-5.99]	0.005^∗^
Administration and business	3.11 [1.65-5.84]	1.98 [0.93-4.24]	0.076
Languages and theoretical studies	4.08 [2.18-7.63]	3.62 [1.68 -7.78]	0.001^∗^
Others	9.91 [3.72-26.43]	5.89 [1.90-18.25]	0.002^∗^

*Place of residence (city)*			
Riyadh	1.00	1.00	
Abha	1.44 [0.85-2.43]	0.90 [0.36-2.23]	0.824
Dammam	0.50 [0.27-0.93]	0.62 [0.29-1.33]	0.223
Jeddah	0.99 [0.53-1.86]	0.85 [0.40-1.79]	0.679

Note: Independent variables for the multiple logistic regression model analysis included age, sex, education, field of study, and place of residence. ^+^Students not able to recognize any one of the foodborne pathogens listed in this study. ^∗^Statistically significant (*p* < 0.05).

## Data Availability

The data sets used and/or analyzed during the current study are available from the corresponding author on reasonable request.

## Abbreviations

*E. coli*: *Escherichia coli*.
